# A new spinosaurid dinosaur species from the Early Cretaceous of Cinctorres (Spain)

**DOI:** 10.1038/s41598-023-33418-2

**Published:** 2023-05-18

**Authors:** Andrés Santos-Cubedo, Carlos de Santisteban, Begoña Poza, Sergi Meseguer

**Affiliations:** 1 Àrea de Cristal·lografia i Mineralogia, Departament de Biologia, Bioquímica i Ciències Naturals, Universitat Jaume I, 12071 Castelló, Spain; 2 Grup Guix, 12540 Vila-real, Spain; 3 Departament de Botànica i Geologia, Universitat de València, 46100 Burjassot, Spain

**Keywords:** Palaeontology, Taxonomy

## Abstract

A new spinosaurid genus and species is described based on the right maxilla and five caudal vertebrae of a single specimen from the Arcillas de Morella Formation (Early Cretaceous) at the locality of Cinctorres (Castellón, Spain). *Protathlitis cinctorrensis* gen. et sp. nov. is diagnosed by one autapomorphic feature as well as by a unique combination of characters. The autapomorphy includes a subcircular depression in the anterior corner of the antorbital fossa in the maxilla. The new Iberian species is recovered as a basal baryonychine. The recognition of *Protathlitis cinctorrensis* gen. et sp. nov. as the first baryonychine dinosaur species identified from the Arcillas de Morella Formation (late Barremian) from the same time as *Vallibonavenatrix cani*, the first spinosaurine dinosaur from the same formation in the Morella subbasin (Maestrat Basin, eastern Spain), indicates that the Iberian Peninsula was home to a highly diverse assemblage of medium-to-large bodied spinosaurid dinosaurs. It seems that spinosaurids appeared during the Early Cretaceous in Laurasia, with the two subfamilies occupying the western part of Europe during this period. Later, during the Barremian–Aptian, they migrated to Africa and Asia, where they would diversify. In Europe, baryonychines were dominant, while in Africa, spinosaurines were most abundant.

## Introduction

In recent decades, scientists have conducted intensive research on spinosaurid evolution, particularly in a probable European origin of the clade^1,2^. Since the discovery of *Baryonyx walkeri*^3^, new species have been described mainly in Western Europe: *Camarillasaurus cirugedae*^4^ and *Vallibonavenatrix cani*^5^ from Spain, *Iberospinus natarioi*^1^ from Portugal, and *Ceratosuchops inferodios* and *Riparovenator milnerae* from the United Kingdom^2^.

The fossil record of the Spinosauridae family comprises fossils from the Cenomanian deposits of North Africa assigned to *Spinosaurus*^6–10^ and of South America to *Oxalaia*^11^, Albian of South America to *Irritator*^12^ and *Angaturama*^11^, Aptian of Southeast Asia to *Ichthyovenator*^13^ and of Africa to *Suchomimus*^14^, Barremian of Western Europe to *Baryonyx*, *Camarillasaurus*, *Ceratosuchops*, *Iberospinus*, *Riparovenator* and *Vallibonavenatrix*^1–5^ and of Southeast Asia to *Siamosaurus*^15^. Recently, Sereno et al.^16^ tentatively assigned *Ceratosuchops* and *Riparovenator* to a single taxon, *C. inferodios*, as the sister taxon to *Suchomimus tenerensis*, and put together *Irritator* and *Angaturama* (they consider *Angaturama* as a junior synonym). Thus, the Spinosauridae family has at least eleven valid genera of spinosaurids, five of them from the Barremian of Western Europe, divided into the subfamilies, Baryonychinae and Spinosaurinae^2^. However, these works are only some contribution to a yet ongoing debate about the nature and paleobiology of South American, African, Asian and European spinosaurids, which is best exemplified in long series of key papers^16–22^.

In Spain, spinosaurid fossils are restricted to the Lower Cretaceous from the Barremian of Teruel and Castellón (Maestrat Basin) and the Barremian–Aptian of Burgos and La Rioja (Cameros Basin) and what may be a tooth from the Barremian of Cuenca (Serranía de Cuenca Basin), but most of the fossils are isolated teeth^23^. In most cases, teeth do not allow determination beyond the subfamily level, and variation in spinosaurid crown ornamentation has uncertain taxonomic value within Spinosauridae and may be influenced by both tooth position and ontogeny^24–26^.

To date, postcranial elements assigned to spinosaurids have been recovered from Teruel (manual ungual phalanx and two isolated vertebrae)^27^, La Rioja (nearly complete hindlimb)^23^ and Castellón (cervical and caudal vertebrae and a nearly complete left tibia)^28^. Additionally, two new species are described based on postcranial elements, *Camarillasaurus cirugedae*^4^ from Teruel and *Vallibonavenatrix cani*^5^ from Castellón. Finally, an isolated maxilla (CPI 477) from the Lower Cretaceous of the Cameros Basin (La Rioja) previously assigned to *Baryonyx walkeri* is now assigned to Baryonychinae indet^29^.

Recent discoveries, such as the fossils from the ANA site (Cinctorres, Spain) described below, might shed light on the knowledge of spinosaurid origins and evolution. The fossils include a partial right maxilla and five caudal vertebrae of a new baryonychine spinosaurid named *Protathlitis cinctorrensis* gen. et sp. nov. from the Arcillas de Morella Formation (late Barremian, Cinctorres, Spain). Finally, we discuss the possible implications of these new taxa for baryonychine diversity and ecology in Spain, and our interpretation indicates the presence of at least two spinosaurid taxa within the Arcillas de Morella Formation, a Baryonychinae and a Spinosaurinae.

## Results

### Geographical and geological setting

**Figure 1:**
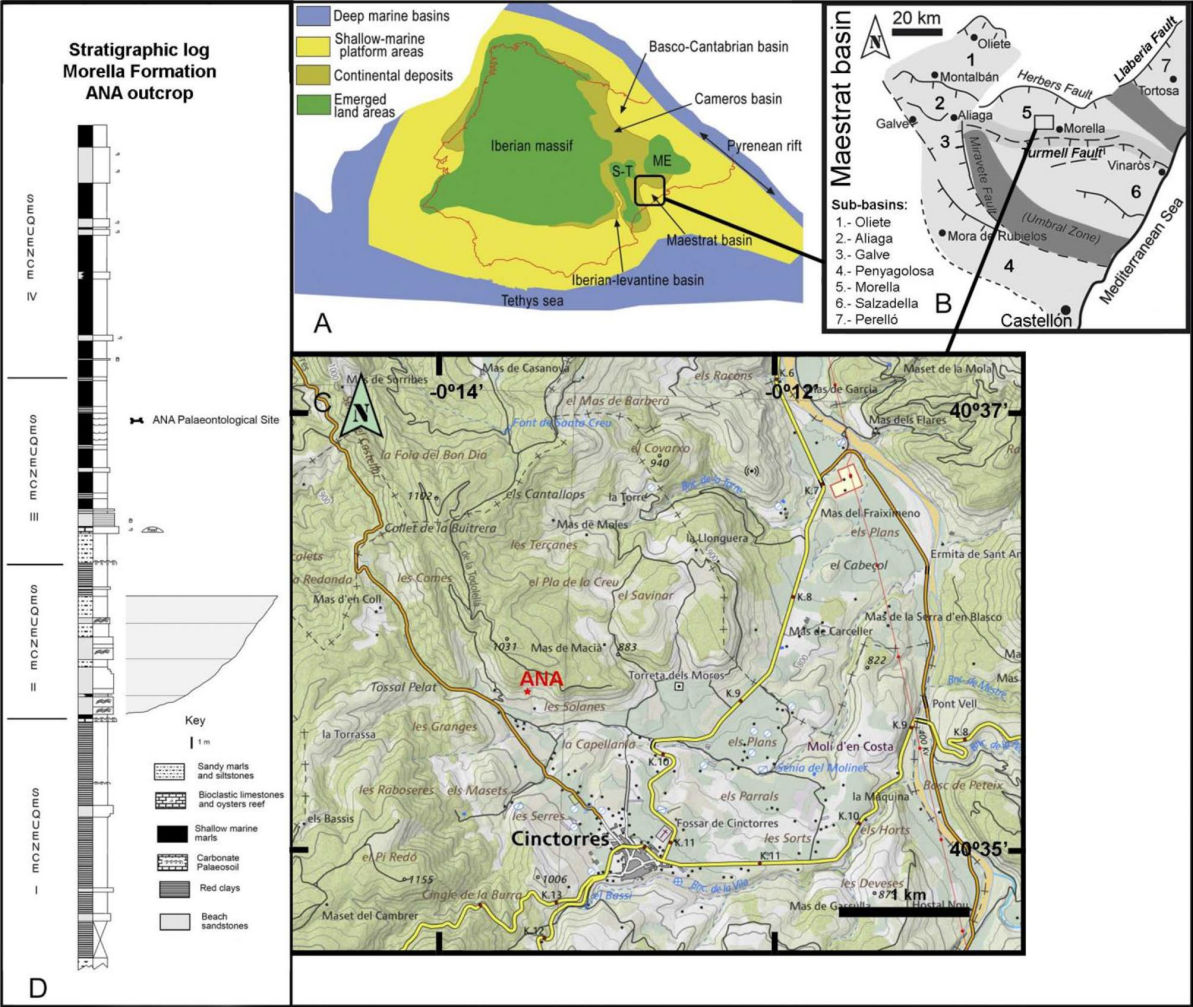
Geographical and geological location of the ANA fossil locality in Cinctorres (Castellón, Spain). (**A**) Palaeogeographic basins of the Iberian Plate during Early Cretaceous, modified from figure in Santos-Cubedo et al.^33^ under a CC BY license 4.0. (**B**) Palaeogeographic sub-basins within the Maestrat Basin and active faults during Early Cretaceous sedimentation, modified from figure in Santos-Cubedo et al.^33^ under a CC BY license 4.0. (**C**) Geographical location of ANA site. Base map from CV50 2019 CC BY 4.0 © Institut Cartogràfic Valencià, Generalitat (https://visor.gva.es/visor/). (**D**) Stratigraphic column for the Arcillas de Morella Formation in ANA site.

The materials studied herein were recovered at the ANA fossiliferous site. This site is located near the village of Cinctorres (Fig. 1). Cinctorres is approximately 100 km northwest of Castellón de la Plana (Castellón province, eastern Spain) and 12 km WSW of the city of Morella. Geologically, they are located in the tectonosedimentary domain of the Linking Zone of the Iberian Range. The ANA fossil site is found in the materials of the Arcillas de Morella Formation. This formation is a stratigraphic unit from the Lower Cretaceous (upper Barremian) that was deposited in the Maestrat Basin, specifically in the Morella subbasin^30^.

This formation, with a maximum thickness of 96 m, is composed of red clay, yellowish-white sandstone, grey marl, limestone and locally conglomerate. These lithofacies are arranged vertically and laterally, forming a set of depositional sequences limited by erosion surfaces. These sequences have a transgressive–regressive character and are composed of minor cycles of the parasequence type, which may present flood limits, subaerial exposure or erosion. The Morella Formation in the ANA fossil site has an average thickness of approximately 80 m, and a maximum of six depositional sequences have been identified in it. Following Bover-Arnal et al.^31^, the Arcillas de Morella Formation has an age of 0.745 M.a., although each of the six depositional sequences has an average age of 0.124 M.a. This is consistent with Milankovitch cycles of short orbital eccentricity of the Early Cretaceous astronomical periodicities^32^. Further analysis of the influence of astronomical parameters on sedimentology is in progress. All lithologies were deposited in flood plains, estuaries and beaches of a delta. The palaeocurrents come from the north and north‒west from an emerged massif located at the centre of the Iberian Trough. This relief separates the Maestrat Basin from the Iberian–Levantine Basin. In the surroundings of Cinctorres where the fossil site is located, the thickness of the Arcillas de Morella Formation is 57 m.

The ANA site was discovered in 1998 by the geologist Ramón Ortí, but it remained unexcavated until 2002, when a palaeontological team formed by members of the Institut de Paleontologia Miquel Crusafont from Sabadell (Barcelona) and the Grup Guix from Vila-real (Castellón) unearthed the first fossil from the locality. The ANA site is located 37 m from the base of the Arcillas de Morella Formation and consists of approximately 2 m of grey‒yellow sandy mudstones containing limonite and goethite crusts and nodules.

### Systematic palaeontology

DINOSAURIA Owen, 1842.

THEROPODA Marsh, 1881.

TETANURAE Gauthier, 1986.

SPINOSAURIDAE Stromer, 1915.

BARYONYCHINAE Charig and Milner, 1986, sensu Sereno et al., 1998.

Genus *Protathlitis* nov.

LSID urn:lsid:zoobank.org:act:58246771-3887-4E7B-BC7F-695CF08FD97F.

Etymology: Protathlitís (Greek, Πρωταθλητής)—“champion”, in reference to the UEFA Europa League title won by Villarreal C.F. in 2021 and as a tribute to the club centenary celebrated in 2023.

Type species: *Protathlitis cinctorrensis*.

*Protathlitis cinctorrensis* sp. nov.

LSID urn:lsid:zoobank.org:act:FDC6CE8E-D932-4A72-9C3A-DF797E32B539.

Etymology: The specific name is taken from the town in which the fossil was discovered, “Cinctorres”.

**Figure 2:**
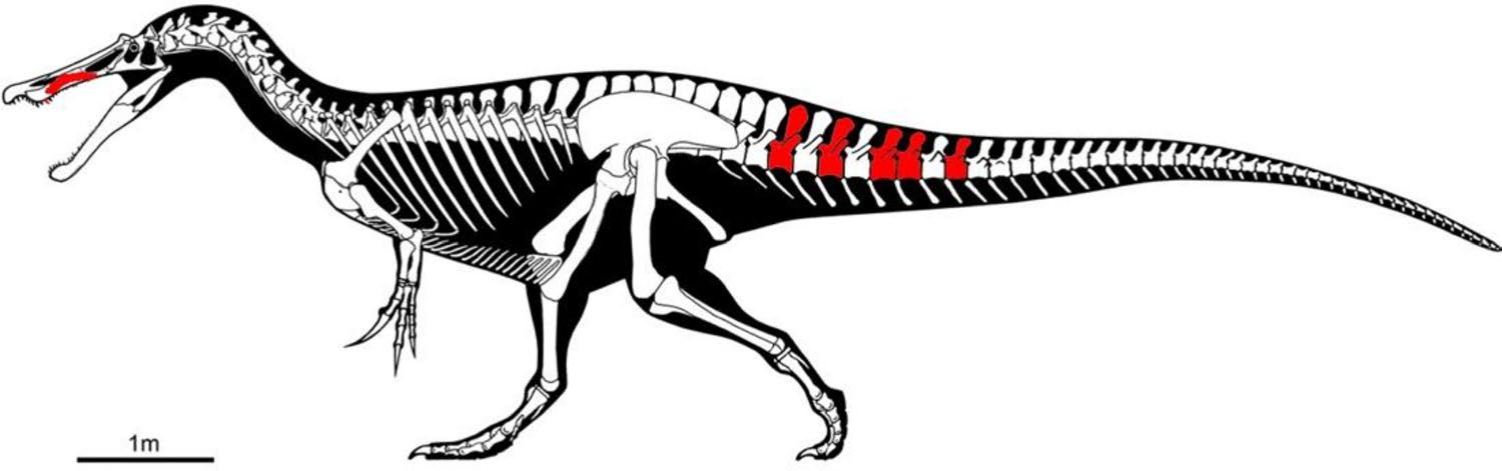
Skeletal diagram showing in red the recovered elements of *Protathlitis cinctorrensis* gen. et sp. nov., modified from figure in Mateus and Estraviz-López^1^ under a CC BY license 4.0.

**Figure 3:**
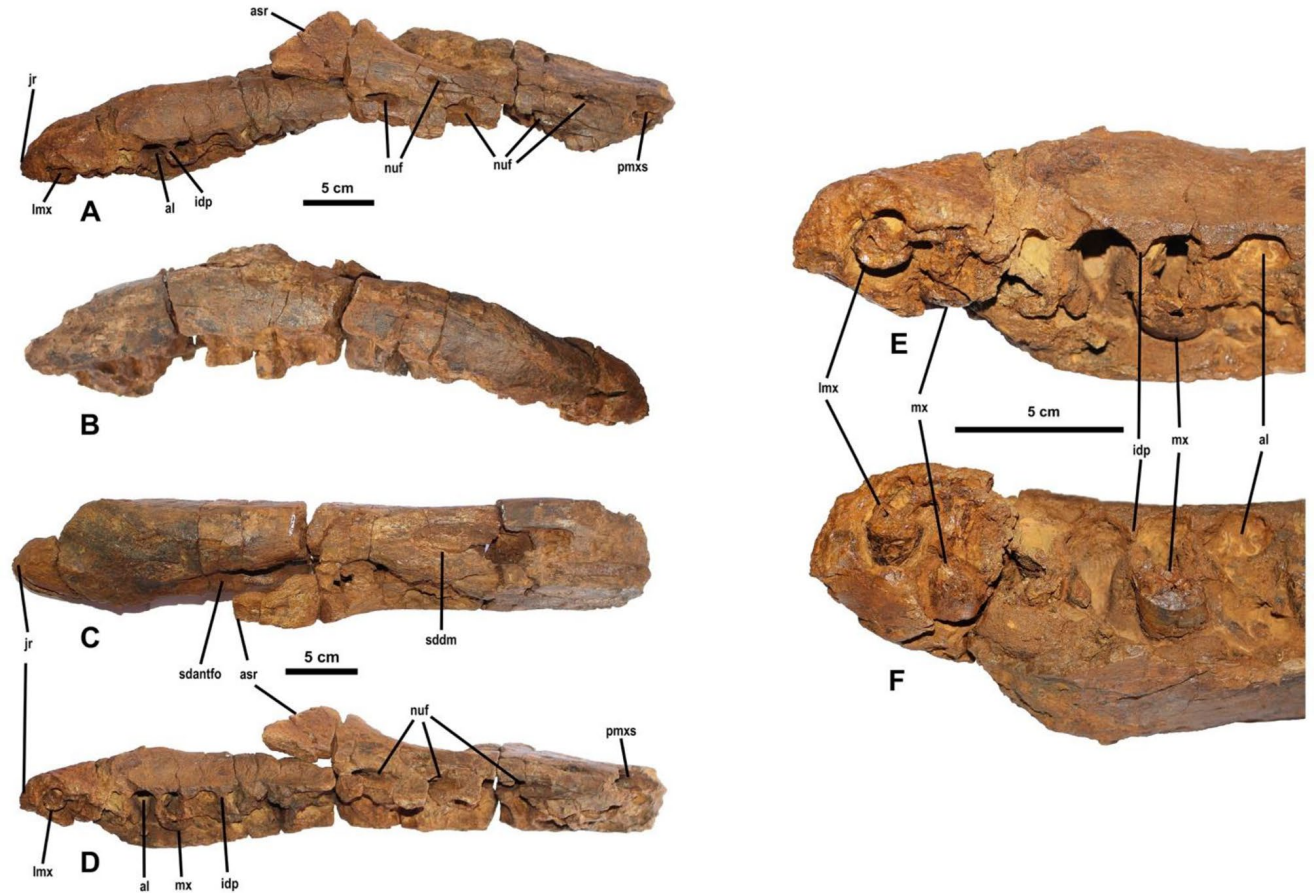
Right maxilla (8ANA-109) of *Protathlitis cinctorrensis* gen. et sp. nov. Labial (**A**), lingual (**B**), dorsal (**C**) and occlusal (**D**) views. Details of four root dental fragments at the tooth row. Occlusal (**E**) and lingual (**F**) views. Abbreviations: al, alveolus; asr, ascending ramus; idp, interdental plates; jr, jugal ramus; lmx, last maxillary teeth; mx, maxillary teeth; nuf, neurovascular foramina; pmxs, promaxillary sinus; sdantfo, subcircular depression in the anterior corner of the antorbital fossa in the maxilla; sddm, subcircular depression in the dorsal margin. Scale bar equals 5 cm.

**Figure 4:**
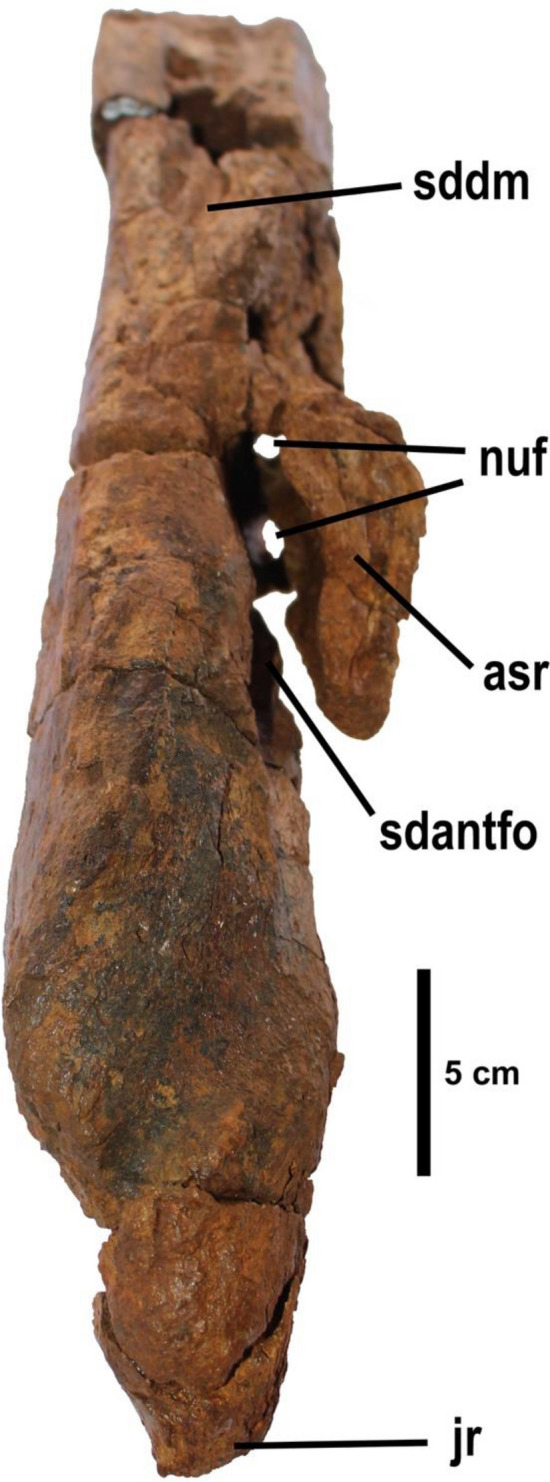
Detail of neurovascular foramina that connect internally into a wide, oval-shaped promaxillary sinus. Abbreviations: asr, ascending ramus; cd; subcircular depression in the anterior corner of the antorbital fossa; jr, jugal ramus; nuf, neurovascular foramina; pmxs, promaxillary sinus; sdantfo, subcircular depression in the anterior corner of the antorbital fossa in the maxilla; sddm, subcircular depression in the dorsal margin. Scale bar equals 5 cm.

**Figure 5:**
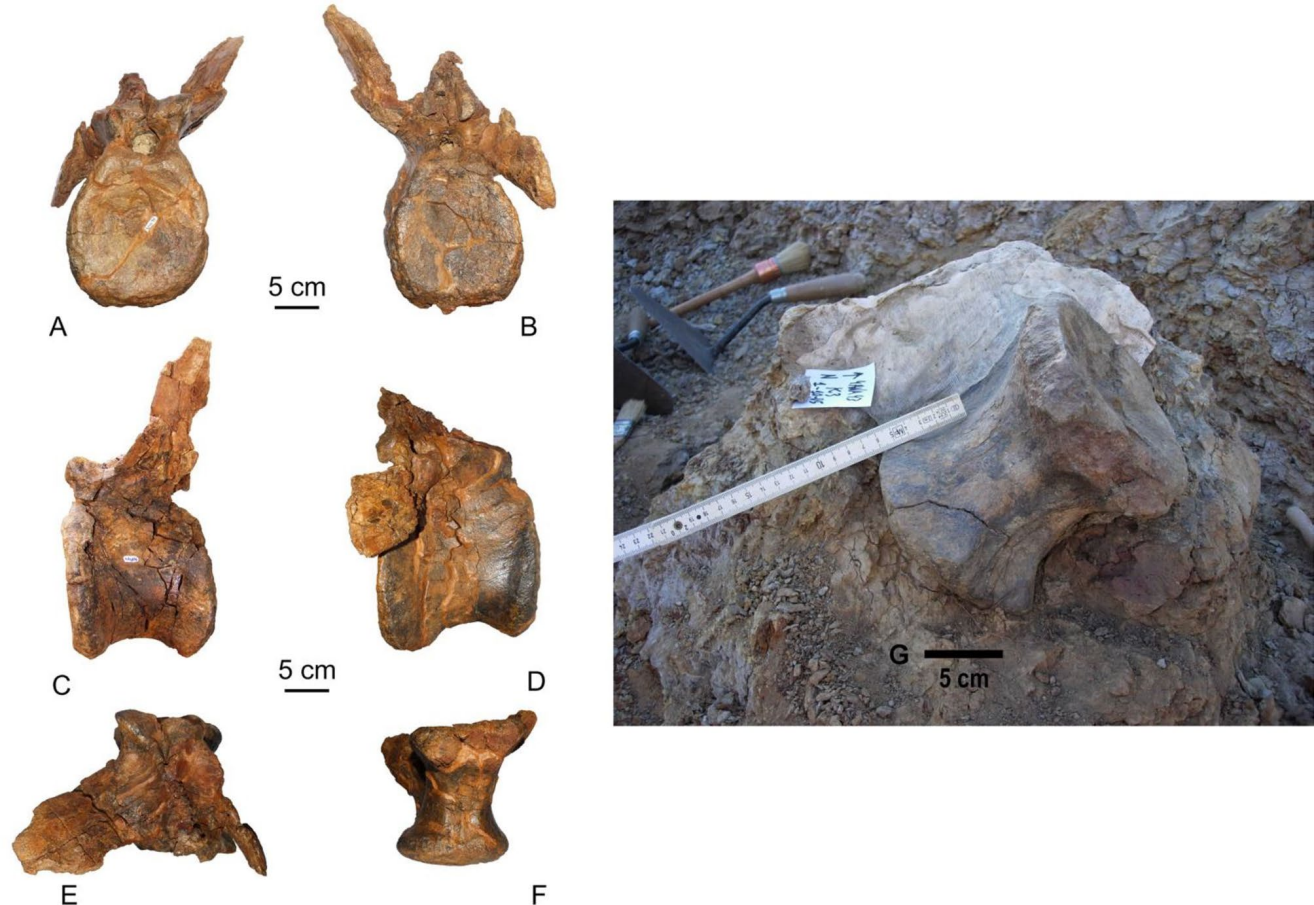
4ANA-76 Anterior caudal vertebrae of *Protathlitis* in anterior (**A**), posterior (**B**), lateral (**C**, **D**), dorsal (**E**) and ventral (**F**). 4ANA-43 Anterior caudal vertebrae of *Protathlitis* in ANA site (**G**). It is noted the grey-yellow sandy mudstones containing limonite crusts covering the fossil and goethite nodule in the lower part of the vertebra. Scale bar equals 5 cm.

**Figure 6:**
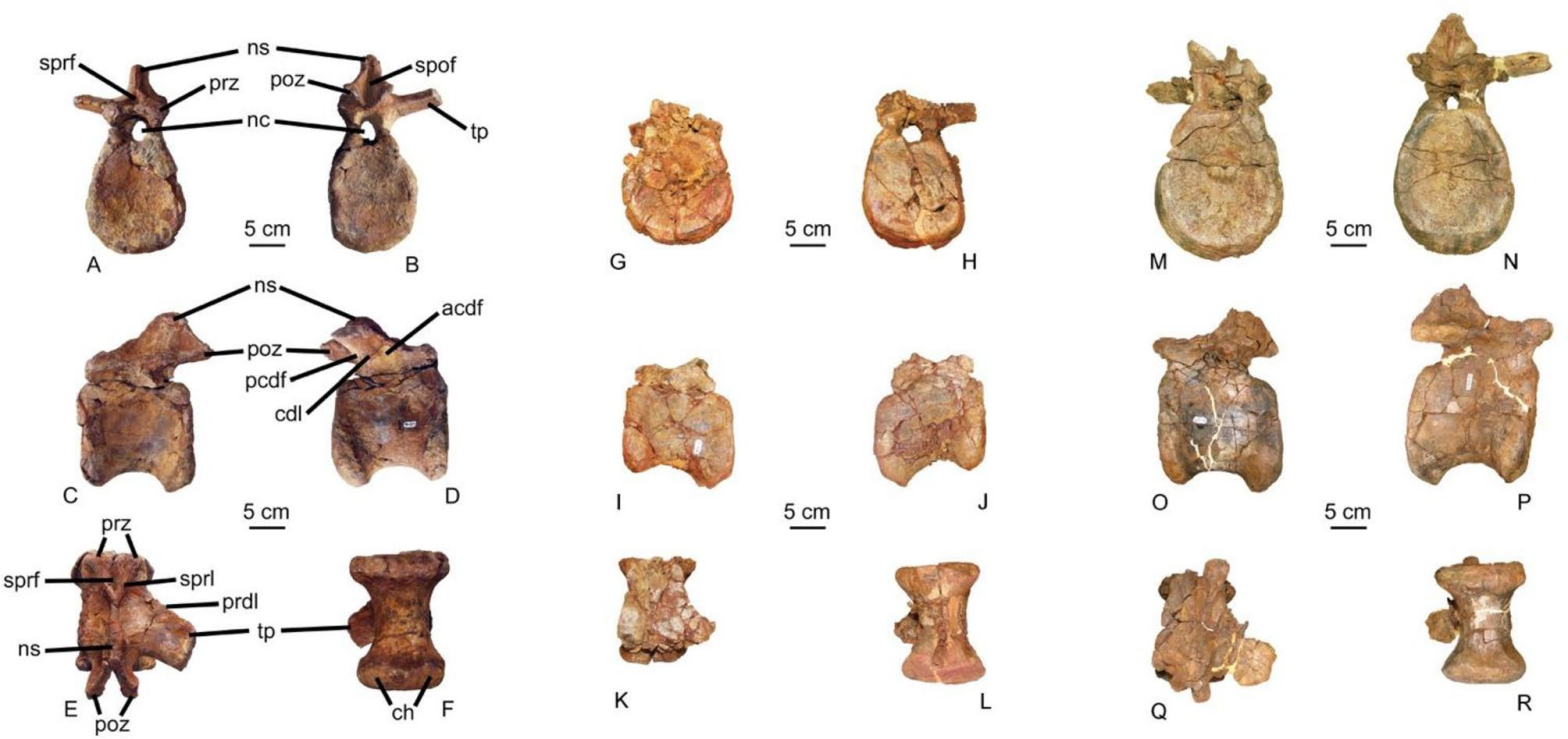
3ANA-83 Mid caudal vertebrae of *Protathlitis* in anterior (**A**), posterior (**B**), lateral (**C**, **D**), dorsal (**E**) and ventral (**F**). Abbreviations: acdf, anterior centrodiapophyseal fossa; cdl, centrodiapophyseal lamina; ch, chevron articulation; nc, neural canal; ns, neural spine; pcdf, posterior centrodiapophyseal fossa; prdl, prezygadiapophyseal lamina; poz, postzygapophysis; prz, prezygapophysis; sprf, spinoprezygapophyseal fossa; sprl, spinoprezygapophyseal lamina; spof, spinopostzygapophyseal fossa; tp, transverse process. 4ANA-69 Mid caudal vertebrae of *Protathlitis* in anterior (**G**), posterior (**H**), lateral (**I**, **J**), dorsal (**K**) and ventral (**L**). 5ANA-78 Mid caudal vertebrae of *Protathlitis* in anterior (**M**), posterior (**N**), lateral (**O**, **P**), dorsal (**Q**) and ventral (**R**). Scale bar equals 5 cm.

Holotype: fragment of a right maxilla (8ANA-109) (Figs. 2, 3, 4) and five caudal vertebrae (3ANA83, 4ANA43, 4ANA69, 4ANA76, 5ANA78) (Figs. 2, 5, 6).

Referred material: 4ANA-11, a left mandibular tooth or a right maxillary tooth, published by Suñer and Santos-Cubedo^34^.

Diagnosis: Baryonychine distinguished by the presence of the following unique autapomorphic feature: subcircular depression in the anterior corner of the antorbital fossa in the maxilla. In addition, *Protathlitis cinctorrensis* gen. et sp. nov. can be distinguished from other baryonychines on the basis of a unique combination of characters: in caudal vertebrae, the transverse process has only two fossae and one buttress, and they are posteriorly oriented; centra with a narrow ventral groove; circular outlines of the articular facets are clearly oval; prezygapophyses and postzygapophysis projected beyond the anterior and posterior rims of the centrum; and no type of element such as hyposphene below the postzygapophysis.

Type locality and type horizon: The ANA site is near Cinctorres (approximately 2 km), Castellón (Spain). The exposed beds belong to the Arcillas de Morella Formation, which is late Barremian (~ 127–126 Ma)^30,31^. Fossils were recovered in grey‒yellow sandy mudstones containing limonite and goethite crusts and nodules that were deposited in a shallow low-energy estuarine environment (Figs. 1, 5). The universal transverse Mercator (UTM) coordinates of the ANA site are 30T YK 734714 4497540.

#### Description

##### Maxilla

8ANA-109 is a nearly complete right maxilla (Figs. 2, 3, 4). It is broken into three separate pieces that fit together. It lacks the anterior part of the maxilla. The maxilla is anteroposteriorly elongate with a length of 430 mm preserved. The maximum width is 71 mm from the medial side of the medial shelf to the lateral wall and is 94 mm in height at its highest point. There are 16 alveoli separated by interdental plates. The maxilla lacks the anterior part, the zone of contact with the premaxilla. Part of the anteromedial process, the ascending ramus and the jugal ramus are preserved. Fragments of four teeth can be distinguished in the most posterior alveolus (Fig. 3). Additionally, some dentine laminae can be observed in other alveoli that form the lower part of the root of some teeth. The medial shelf is broken.

The ventral margin is straight and tapers anteriorly with respect to the distal part in ventral view. The dorsal margin has two subcircular depressions, one situated in the anterior corner of the antorbital fossa (Fig. 3).

The lateral surface of the maxilla is clearly dorsoventrally convex. The lateral wall is pierced by numerous neurovascular foramina arranged in two rows. The lower one is very deep and bears three foramina with an elliptical outline. From each foramen, a groove runs anteroventrally. These grooves are straight or slightly parabolic and become deeper near the foramina. They penetrate the lateral wall of the ramus posterodorsally (Fig. 4). Among these foramina, a dorsally positioned row of two neurovascular foramina running anteroposteriorly can be distinguished. These holes are smaller. Additionally, they are elliptical in outline, a groove runs anteroventrally, becomes deeper near the foramina and penetrates the lateral wall of the ramus posteriorly. Both rows of foramina connect internally into a wide, oval-shaped canal (Figs. 3, 4). This canal runs anteroposteriorly and laterally to the length of the ramus and is parallel to the row of teeth. It originates on a lateral groove from the anterior medial wall of the antorbital fenestra. This canal is the promaxillary sinus^35^.

The maxillary alveoli extend far dorsally, but they are not totally vertical. They are oriented slightly lateral to the external wall of the maxilla. These alveoli are narrower anteriorly, and they become subquadrangular posteriorly. The interdental plates with the continuous interdental bone (sensu Currie^36^) have an hourglass shape in ventral view. Four of the most posterior alveoli retain part of the root of the teeth, decreasing in size. The cross-section of these roots is elliptical. The last of the roots shows the lingual margin not preserved. It has been resorbed to let the replacement tooth migrate labially (Fig. 3), as is observed in other baryonychines^29^.

##### Vertebrae

The identification of the position of the isolated vertebrae in the vertebral column is difficult. In the following description, it is assumed that the vertebral column included forty-one caudal vertebrae or more. This is the general condition in basal tetanurans^37^. The centra of the vertebrae are fused to the neural arch, suggesting that the material belongs to a subadult or an adult form.

The centra of the five anterior vertebrae are well preserved. They are spool-shaped with the side faces lightly compressed. They are amphicoelous, with the proximal face slightly more concave than the distal face. All of the recovered vertebrae are anterior to the “transition point”, defined by the last caudal vertebra with transverse processes^1^ and the first vertebra with elongated prezygapophysis^38^ (usually occurring between Ca17-Ca19). The shape of the centra and the arrangement of the transverse processes suggest that the vertebrae recovered in the ANA site are between positions Ca3 and Ca10.

In the most proximal vertebrae (4ANA43, 4ANA76) (Fig. 5; Table 1), the total height of the centrum is slightly smaller than its length, and the centrum is longer than it is high. The shape of the articular faces is oval with the major axis in the dorsoventral direction. 4ANA43 shows a protuberance in the central part of the two articular faces of the centrum.

**Table 1:** ANA vertebrae dimensions.

	Length	Total height	Total width	Anterior centrum height/posterior centrum height	Anterior centrum width/posterior centrum width
4ANA43	160	205	–	153/156	129/130
4ANA76	156	265	–	150/150	131/132
5ANA78	150	255	155	133/137	114/110
3ANA83	136	225	135	115/111	107/99
4ANA69	135	116	113	112/105	106/96

	Anterior neural canal height/posterior neural canal height	Anterior neural canal width/posterior neural canal width	Lateral process height	Lateral process width	
4ANA43	31/–	–	–	–	
4ANA76	32/32	28/20	177	70	
5ANA78	31/33	34/21	–	64	
3ANA83	32/39	35/22	–	51	
4ANA69	22/23	29/29	–	–	

Measurements are in millimetres.

In lateral view, the distal ventral edge of the centrum is located below the proximal ventral edge, while the distal dorsal edge is rounded forwards to the junction with the neural arch. In the dorsal region of the centrum, just below the lateral processes, is the neurocentral suture, with a marked depression in 4ANA43 (Fig. 5). The more proximal vertebra has a deeper depression dorso-distally in both side faces, just below the neurocentral suture. This depression corresponds to the posterior region of a dorsal protrusion that is observed within the neural canal.

The distal articulation facets of the chevrons are more developed anteriorly than distal facets. There is a groove that divides both of them and continues anteriorly. The anterior facets are not as marked or differentiated as the distal facets. The ventral groove is shallower and narrower anteriorly. In the proximal region, there is a rounded and less developed crest that is located in the midline of the groove, which extends distally to approximately half the centrum.

The transverse processes are not completely preserved, except in 4ANA76 (Fig. 5), which maintains the left process, although it is deformed. The base of the processes is located slightly behind the midpoint of the vertebral centrum and is quite robust in section but flat and wide distally. The branch of the transverse process is directed slightly posterior and dorsally. Ventrally, the processes present a support ridge that is broad and slightly pronounced, which extends to half of their length.

The prezygapophysis of the anterior vertebrae is eroded. In 4ANA76, the facet joint of the right prezygapophysis is directed dorso-medially. In the prezygapophysis, there are well-developed spinoprezygapophyseal laminae. These laminae bind medially near the midpoint of the centrum, where the base of the neural spine arises. Among these laminae, there is a deep and narrow fossa. From the prezygapophysis, there are laterally prezygadiapophyseal laminae. They are bound to the proximal region of the transverse processes. The binding area of the process is located ahead of the union zone between the spinoprezygapophyseal laminae, forming a slightly concave lateral surface. This area is medially deeper near the laminae.

The postzygapophyses of both vertebrae are eroded, and they do not allow observation of the facet joint. The basis of these is located in front of the distal edge of the centrum, and the angle between them is less than that between the prezygapophyses. At the centre of the postzygapophysis, a deep and narrow depression that extends dorsally is observed.

The neural spine is not complete in any of the vertebrae recovered. The base of the spine arises proximally at the junction of the spinoprezygapophyseal laminae, and caudally, it does not exceed the distal edge of the centrum.

The centra of the middle caudal vertebrae (3ANA83, 4ANA69, 5ANA78) (Fig. 6; Table 1) have the same shape as the proximal caudal ones. The length of these vertebrae is greater than their height, as in the case of the proximal caudal ones, they are also amphicoelous, and the articular faces are oval with the dorso-ventral axis greater than the medial–lateral axis.

In lateral view, the distal ventral edge is not as low as in the proximal caudal vertebrae, and the depression of the neurocentral suture is not as marked. In these vertebrae, a deep depression was not observed in the 4ANA43 vertebra.

The articulation facets with the chevrons are more pronounced in the distal region of the vertebrae, and as in the case of the anterior vertebrae, and the facets are less marked in the proximal face. Ventrally, there is a slightly marked groove, but there is not a medial crest.

None of these vertebrae maintain complete transverse processes. The basis of the processes is long antero-posteriorly. The proximal edge arises near the anterior end of the centrum. The lamina of the process is thin in the dorsoventral direction, but in the 5ANA78 specimen, a less developed support crest is observed. The processes are directed posteriorly, and they are slightly dorsal.

The prezygapophysis is badly eroded, and the articular facet is not perceived. As in the case of the proximal vertebrae, the spinoprezygapophyseal laminae are highly developed, and they are joined together near the base of the prezygapophysis, forming a narrow but deep fossa anteriorly. In the 5ANA78 vertebra, the right prezygadiapophyseal lamina forms a thin ridge on the anterior edge of the process, as in the case of the anterior vertebrae forming a large lateral concave surface to the spinoprezygapophyseal lamina.

The postzygapophyses are long and protrude slightly behind the distal edge of the centrum. The articulation facets are large, and they are lateroventrally oriented at an angle similar to that of the anterior vertebra. Dorsally to the postzygapophysis, a deep and elongated depression is observed that is directed towards the neural spine.

The neural spine is not complete in any of the medial vertebrae. The base is slightly shorter in the anterior–posterior direction than in the case of the anterior vertebrae, and it originates from the joining of the spinoprezygapophyseal lamina, which is anterior to the midline of the centrum.

### Phylogenetic results

**Figure 7:**
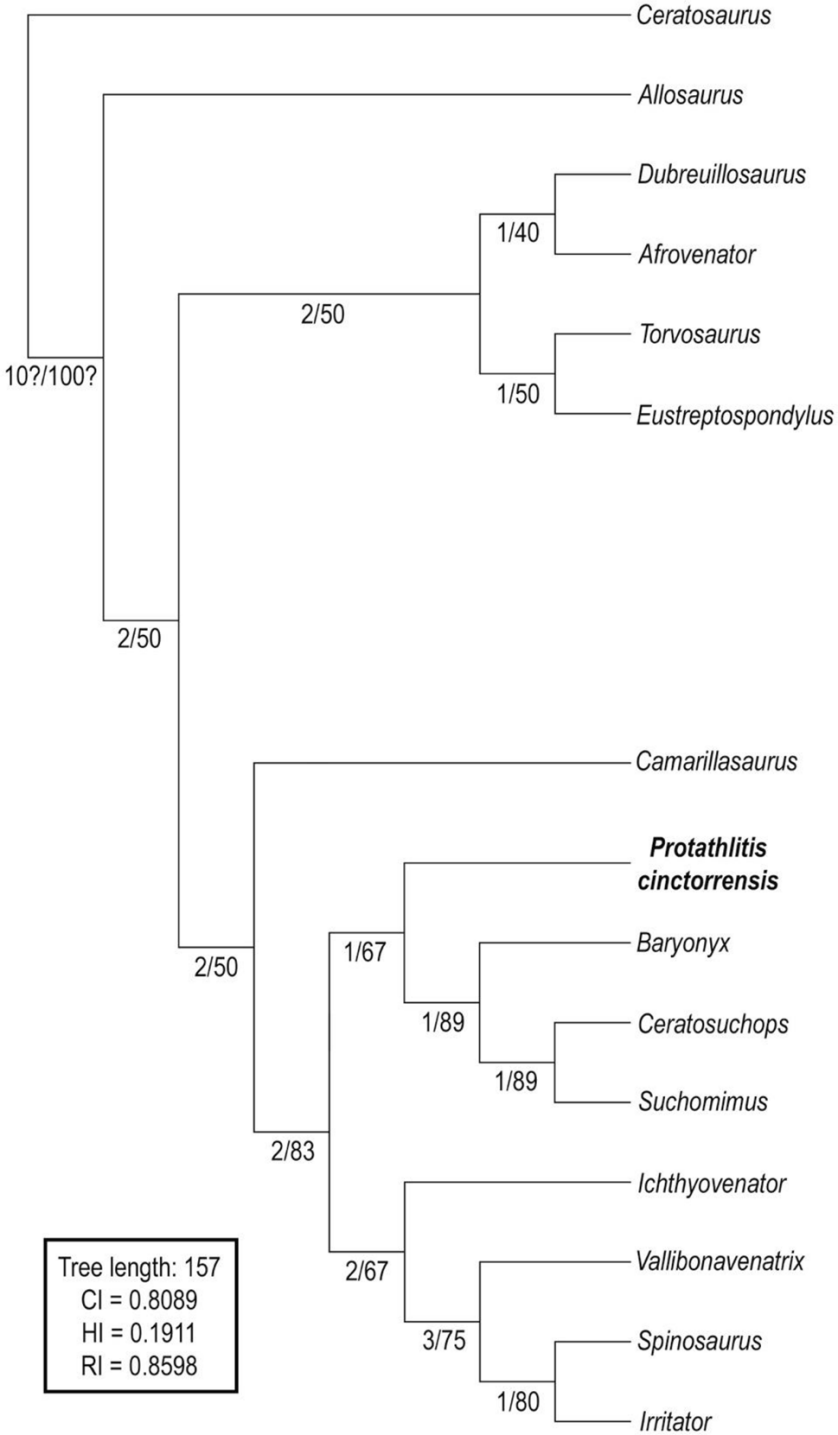
Phylogenetic relationships of *Protathlitis cinctorrensis* gen. et sp. nov. One MPT (Most Parsimonious Tree) recovered from dataset of 15 OTUs with 157 steps (CI = 0.8089, HI = 0.1911, RI = 0.8598). Notes: Numbers at the left of the node indicate, first, the decay index (Bremer support from 369 trees, cut 0) and, second, the Relative Bremer support (from 143 trees, cut 0). The phylogenetic position of *Protathlitis cinctorrensis* is indicated in bold.

Using the phylogenetic analysis program TNT^39^, we performed a “traditional search” with tree bisection reconnection. Wagner trees with a random seed of 1 and 9,999 replicates with 10 trees saved per replication were used. All characters were equally weighted and treated as unordered. TNT revealed 9,386,515 rearrangements. As a result, one most parsimonious tree (MPT) was recovered (Fig. 7) with a length of 157 steps, a consistency index (CI) of 0.8089, a homoplasy index (HI) of 0.1911, and a retention index (RI) of 0.8598 (the ‘describetree’ command in PAUP 4.0a build 167^40^).

In the resulting tree, *Protathlitis cinctorrensis* is tentatively positioned as the most basal baryonychine. Our phylogenetic analysis is consistent with the definition of Spinosauridae (with *Camarillasaurus*), Ceratosuchopsini (*Suchomimus* + *Ceratosuchops*), Spinosaurinae, Vallibonavenatrix (*Spinosaurus* + *Irritator*) and *Spinosaurus* + *Irritator*. However, in our analysis, Baryonychinae is supported only by one apomorphic character: Ch. 57 (0 → 1) 30 dentary teeth (unambiguous).

*Protathlitis cinctorrensis* is recovered as a basal baryonychine (Fig. 7). Unambiguous synapomorphies include character 14, which corresponds to a: (14) Maxilla, subcircular depression in the anterior corner of the antorbital fossa present.

Mateus and Estraviz-López^1^ described a new Spinosauridae dinosaur, *Iberospinus natarioi*. They pointed out that the fossil shows some characteristics of baryonychines, suggesting a closer relation to the group but placing it outside of both the Baryonychinae and the Spinosaurinae. This suggests that *Iberospinus* would be a sister taxon to *Camarillasaurus* (outside both subfamilies), or perhaps both taxa are the same (synonymy). Future research should focus on this topic.

## Discussion

### Maxilla

8ANA-109 shares with Spinosauridae the proportionally larger medial shelf (anteroposteriorly and mediolaterally) compared with the rest of tetanurans^29^. Additionally, it shares with this family the posterior decrease in the size of the alveoli, the conidont dentition and the veined/anastomosed enamel texture^29^.

Sereno et al.^14^ recognized some features that differentiate Baryonychinae from Spinosaurinae. The former has curved tooth crowns, teeth with fine serrations, external naris retracted to the first half of the maxillary tooth row and an elevated number of maxillary teeth (22 in *Suchomimus*). In contrast, Spinosaurinae has unserrated teeth; tooth crowns slightly curved or straight; external naris retracted farther caudally and fewer maxillary teeth (12 well-spaced maxillary teeth in *Spinosaurus* (MSNM V4047) and 11 in *Irritator*).

The elevated number of maxillary teeth (almost 16), curved crown and teeth with fine serrations situate 8ANA-109 as a member of Baryonychinae. In this subfamily, there are four valid genera, *Baryonyx*, *Ceratosuchops*, *Iberospinus* and *Suchomimus*^16^. Only *Baryonyx*^3^ and *Suchomimus*^16^ have maxillary remains. The 8ANA-109 fossil differs from *Suchomimus*^16^ because it has a subcircular depression in the anterior corner of the antorbital fossa that is absent in the African dinosaur. This character in not preserved in *Baryonyx*^3^.

The shape of the alveoli is subcircular, as in other spinosaurids, but differs from the more elliptical alveoli in a fossil maxilla (CPI 477) from Igea (Spain)^29^.

8ANA-109 shows two replacement teeth at different stages of development within the same alveolus, as is seen in other theropods, such as *Irritator*, *Oxalaia*, *Iberospinus* and the CPI 477 maxilla^29^.

Maxilla of some known spinosaurids are approximately 42–46 cm (BMNH R9951—*Baryonyx walkeri*; MNBH EGA 1—*Spinosaurus* sp.; MNN GDF501—*Suchomimus tenerensis*)^3,9,14,16^, and it has been calculated that these dinosaurs would have a length of 10-12 metres. Therefore, according to the dimensions of the *P. cinctorrensis* maxilla (43 cm), it is estimated that it was a large-sized theropod (between 10-11 metres).

### Vertebrae

The vertebral centra have the typical spool shape that is present in most theropods, with a U-shaped cross section, unlike in the case of *Baryonyx walkeri*, where the caudal vertebral centra are highly compressed^3^. Additionally, in the ANA theropod specimen, there is no type of element such as hyposphene below the postzygapophysis, as in *B. walkeri*^3^. A well-developed infradiapophysial lamina and a deep infradiapophysial fossa are present in the neural arch in *Baryonyx* but not in *Protathlitis*.

Caudal vertebrae from the ANA site differ from those of *Riparovenator milnerae*^2^ in the shape of the neural arch. In *R. milnerae*, transverse processes start from the prezygapophysis, widely occupy the length of the centrum, and maintain their width transversally. Additionally, the prezygapophysis is elevated from the centra, the postzygapophysis is more developed posteriorly, and at the base of the transverse processes, the centrodiapophyseal lamina is absent in the anterior neural arches.

Additionally, neural arches of *Spinosaurus*^41^ are very different from those from the specimen from the ANA site, with a notably complex array of vertebral laminae and fossae in the proximal caudal vertebrae. In *Spinosaurus*, the transverse process has three centrodiapophyseal fossae and two centrodiapophyseal laminae, but in the ANA specimen, the transverse process has only two fossae and one buttress. Additionally, the anterior caudal vertebrae of *Spinosaurus* exhibit wide and shallow ventral grooves that differ from the centra of the ANA specimen, which have a narrow ventral groove. Additionally, the transverse processes in *Spinosaurus* are projected perpendicular to the centra, while in *P. cinctorrensis*, they are posteriorly oriented.

*Ichthyovenator*^13^ caudal vertebrae are clearly different. They have robust and elongate transverse processes that extend posterolaterally and dorsally. The first transverse process is sigmoid in shape in dorsal view. Prezygodiapophyseal and anterior centrodiapophyseal laminae are well marked and define a deep prezygapophyseal centrodiapophyseal fossa unknown in other theropods^13^.

The caudal vertebrae of *Siamosaurus*^15^ (Phuwiang spinosaurid B), *Camarillasaurus*^4^ and *Iberospinus*^1^ are all similar^15^. These specimens mainly differ from *Protathlitis* in that they have two buttresses and three fossae below the transverse process of the caudal vertebrae.

*Iberospinus*^1^, *Suchomimus*^14^, *Spinosaurus*^41^ and possibly *Camarillasaurus*^4^ and *Ichthyovenator*^13^ have anterodorsally inclined prezygapophyses in lateral view that do not project beyond the anterior rim of the centrum, unlike in ANA fossils.

*V. cani*^5^ is different in size, and ANA fossils are at least twice as large as from Vallibona. Another difference is the circular outlines of the articular facets that are approximately as wide as they are high in *V. cani*^5^, while in a similar position (Ca9), those of *P. cinctorrensis* (3ANA83, 4ANA69, 5ANA78) are clearly oval. The same occurs with the neural canal. The anterior and posterior edges are at the same level in Vallibona specimens, while in ANA vertebrae, the posterior edge is lower than the anterior edge. Postzygapophyses are short, projecting only slightly beyond the level of the posterior articular facet, in contrast with those from ANA, which clearly surpass the posterior articular facet. Finally, the transverse process of *P. cinctorrensis* is longer.

*Suchomimus tenerensis*^14,16^ vertebrae are similar to those of *Protathlitis*, but differ because the prezygapophysis is more dorsally projected and the postzygapophysis is shorter (slightly projecting beyond the posterior rim of the centrum) than in *P. cinctorrensis*.

The ventral surface in the anterior caudal vertebrae has a groove in ANA fossils, as in *Camarillasaurus*^4^, *Vallibonavenatrix*^5^ and *Spinosaurus*^41^. This differs in *Baryonyx*^3^, *Suchomimus*^14^ and *Ichthyovenator*^13^ with a flat ventral surface.

Finally, the anterior caudal vertebra from the ANA site has a centrum with a height/length ratio below 1, indicating that it is longer than it is high. This is the same in *Baryonyx*^3^ (BMNH R9951 CaB) and *Suchomimus*^16^ (MNBH GAD 71 Ca1 and MNBH GAD 85 Ca2). This is different in *Iberospinus*^1^ (ML1190-15 Ca4), *Ichthyovenator*^13^ (BK 10-02 Ca1 and BK 10-03 Ca2), *Riparovenator*^2^ (IWCMS 2020.447.5 Ca9) and *Spinosaurus*^41^ (FASC-KK 11,888 Ca2 and Ca4), in which the most anterior caudal vertebrae are higher than they are long (ratio above 1).

## European evolution

Spinosaurids have been recovered in the western part of Europe, mainly in Portugal, Spain and the United Kingdom^1,2,5,22,29^. However, the most common fossils are teeth^23^. In the Lower Cretaceous formations, fossils are present from the upper Hauterivian to Aptian^27^. The basal spinosaurids *Camarillasaurus*^4^ and *Iberospinus*^1^ are Barremian in age, similar to *Baryonyx*^3^, *Ceratosuchops/Riparovenator*^2^ and *Protathlitis* as members of baryonychines and *Vallibonavenatrix*^5^ as a spinosaurine (for Barker et al.^2^, *V. cani* is located basal to the split of the spinosaurid subfamilies). *Suchomimus*^16^ (baryonychine) is Aptian, and the rest of spinosaurines are *Ichthyovenator*^13^ (Aptian), *Spinosaurus*^41^ (Cenomanian) and *Irritator*^11,12^ (Albian). It seems clear that the origin of the family may be in western Europe, as suggested by Milner^42^, Buffetaut^43^, Isasmendi et al.^23^ and Barker et al.^2^, among others. It also seems clear that in the western part of Europe during the Barremian, at least two different taxa of spinosaurids coexisted^23^. All the findings produced to date seem to indicate that the group originated in western Europe during the Late Jurassic or Early Cretaceous and then expanded to Africa and Asia. For example, in the Iberian Peninsula, spinosaurids are still the most common theropods in the Lower Cretaceous formations (from the upper Hauterivian to Aptian^27^). Another example is that both baryonychines and spinosaurines are documented in different areas, such as the Cameros and Maestrat basins^23^ during the Early Cretaceous (Barremian–Aptian). During the Barremian–Aptian, spinosaurids moved from Europe to Africa and diversified there during the Aptian-Cenomanian period. Several migratory routes have been suggested between Gondwana and Laurasia during the Early Cretaceous, such as the ‘Apulian route’^44,45^ or during the Late Cretaceous^46^ based on ichnites and skeletal remains.

Therefore, a possible scenario would be the following: spinosaurids appeared during the Early Cretaceous in Europe and diversified, appearing as members of the two subfamilies and occupying a large part of western Europe. Later, during the Barremian–Aptian, they migrated to Africa and Asia, where they diversified again. In Europe, baryonychines were dominant, while in Africa, spinosaurines were the most abundant.

Finally, previous works suggested that spinosaurids inhabited inland areas far from the coast or freshwater environments with marine influence or next to coastal zones^23,27,47^. In the case of ANA dinosaurs, they inhabited areas next to coastal zones.

## Conclusions

This work describes and discusses the type specimen of the spinosaurid baryonychine *Protathlitis cinctorrensis* gen. et sp. nov. from the Arcillas de Morella Formation, late Barremian (Early Cretaceous) of Cinctorres (Castellón, Spain). The holotype is described based upon a cranial element (fragment of a right maxilla: 8ANA-109) (Figs. 2, 3, 4) and postcranial elements (five caudal vertebrae: 3ANA83, 4ANA43, 4ANA69, 4ANA76, 5ANA78) (Figs. 2, 5, 6; Table 1) of a large individual (10–11 m).

These fossils belong to Spinosauridae because they have a larger medial shelf, alveoli decreasing in size posteriorly, conidont dentition and veined/anastomosed enamel texture. Additionally, they belong to Baryonychinae because they have curved tooth crowns, teeth with fine serrations, external naris retracted to the first half of the maxillary tooth row and an elevated number of maxillary teeth. Inside this subfamily, one autapomorphy supports the validity of the type species (subcircular depression in the anterior corner of the antorbital fossa in the maxilla). In addition, *Protathlitis cinctorrensis* gen. et sp. nov. can be distinguished from other baryonychines on the basis of a unique combination of characters: in caudal vertebrae, the transverse process has only two fossae and one buttress, and they are posteriorly oriented; centra with a narrow ventral groove; circular outlines of the articular facets are clearly oval; prezygapophyses and postzygapophysis projected beyond the anterior and posterior rims of the centrum; and no type of element such as hyposphene below the postzygapophysis. Regardless of the phylogenetic analysis carried out, *P*. *cinctorrensis* is clearly nested as a basal baryonychine (Fig. 7).

The establishment of this new European species seems to confirm that spinosaurids appeared during the Early Cretaceous in Laurasia, with the two subfamilies occupying the western part of Europe. Later, during the Barremian–Aptian, they migrated to Africa and Asia, where they would diversify. In Europe, baryonychines were dominant, while in Africa, spinosaurines were the most abundant.

## Methods

### Phylogenetic analysis

A phylogenetic analysis was conducted using a modified version of the Sereno et al.^16^ dataset (Supplementary File S1) based on a 120-character list (49% cranial, 51% postcranial). We revised the dataset in its matrix, and we added one missing vertebral character to the specimen *Baryonyx walkeri*^3^ (character 82: Caudal vertebrae, anterior, morphology of ventral surface changed from (?) to flat (0)). The matrix includes 15 × 148 operational taxonomic units (OTUs). For *Protathlitis cinctorrensis* gen. et sp. nov., twenty-five (15 cranial and 10 postcranial) characters were scored from the available information from the specimen (holotype). This modified matrix was analysed using TNT v.1.5 software^39^. All characters were equally weighted and treated as unordered. Using TNT software, we calculated the ‘Bremer Support’, and we identified which synapomorphies supported each node. Only unambiguous synapomorphies were calculated, and branches with no possible support were collapsed ("rule 3"). This makes the results more conservative.

*Ceratosaurus*, as a tetanuran outgroup, and *Allosaurus*, as a non-spinosauroid theropod, were employed as constraints.

## Supplementary Information

Supplementary Information.

## Data Availability

All data generated or analysed during this study are included in this published article and its supplementary information files.
